# Risk factors of left ventricular diastolic dysfunction in maintenance hemodialysis patients

**DOI:** 10.1186/s12882-023-03220-3

**Published:** 2023-06-12

**Authors:** Ying Lei, JinYan Tong, YanYan Su, YuQuan Wang, BiXian Zhong, Qi Wang, YunFang Zhang

**Affiliations:** Department of Nephrology, Huadu District People’s Hospitalof Guangzhou, Southern Medical University, Guangzhou, 510800 China

**Keywords:** Hemodialysis, Echocardiography, Cardiac structure, Left ventricular diastolic dysfunction

## Abstract

**Objective:**

To investigate the risk factors of left ventricular diastolic dysfunction in maintenance hemodialysis (MHD) patients.

**Method:**

We retrospectively collected data from 363 hemodialysis patients who were on dialysis for at least 3 months at January 1, 2020. According to the echocardiogram results, these patients were divided into left ventricular diastolic dysfunction (LVDD) group and non-LVDD group. The differences in basic data, cardiac structure and functiona between the two groups were analyzed. Logistic regression analysis was used to analyze the risk factors of cardiac diastolic dysfunction in MHD patients.

**Results:**

Compared with the non-LVDD group, patients in the LVDD group were older, with an increased proportion of coronary heart disease, more prone to chest tightness, shortness of breath. Simultaneously, they had a significantly increased (*p* < 0.05) proportion of cardiac structural abnormalities such as left ventricular hypertrophy, left heart enlargement and systolic dysfunction. Multivariate logistic regression analysis showed that the risk of LVDD was significantly increased in elderly MHD patients older than 60 years (OR = 3.86, 95%CI 1.429–10.429), and left ventricular hypertrophy was also significantly associated with LVDD (OR = 2.227, 95% CI 1.383–3.586).

**Conclusion:**

According to research, both age and left ventricular hypertrophy are risk factors for LVDD in MHD patients. It is recommended that early intervention for LVDD should be implemented to improve the quality of dialysis and reduce the incidence of cardiovascular events in MHD patients.

## Background

Cardiovascular disease (CVD) is a common comorbidity in chronic kidney disease (CKD) patients [1], it is also the leading cause of death in patients undergoing maintenance hemodialysis [2]. Left ventricular diastolic dysfunction (LVDD) is commonly observed in CKD patients and occurs in the early stages of heart disease [3]. At the same time, progressive diastolic dysfunction is independently associated with a higher probability of death and has a similar impact on survival and death [3]. Early detection and intervention of LVDD can reduce the incidence of cardiovascular events and improve the prognosis of MHD patients [4]. In this study, we used the basic information of MHD patients combined with the results of echocardiography to find out the relevant risk factors affecting the diastolic function of the heart and to evaluate their impacts on the cardiac function, providing insights and guidance for clinical diagnosis and functional management of cardiovascular diseases in MHD patients.

## Materials and methods

This was a retrospective cross-sectional study in which all patients that underwent dialysis for at least 3 months at January 1, 2020 were included.

### Inclusion criteria

age ≥ 18 years, regular hemodialysis patients whose hemodialysis age ≥ 3 months, consent to the study and signed informed consent.

### Exclusion criteria

acute myocardial infarction three months before enrollment. Severe heart valve disease or rheumatic heart disease and patients who disagree with the study.

The information we collected included age, gender, hemodialysis age (months), the primary disease of kidney and complications, such as hypertension, diabetes, coronary heart disease and heart failure. Dialysis frequency and average ultrafiltration capacity per time were collected. Venous blood was collected before dialysis, and we also collected the test items involving hemoglobin, serum creatinine, uric acid, albumin, calcium, phosphorus, parathormone, brain natriuretic peptide(BNP) and cardiac troponin I (cTnI).

Echocardiography examination was performed on non-dialysis days by an experienced sonographer using a Philips color doppler instrument (CX50). Tissue Doppler methods were used to assess diastolic function of the heart.We measured right ventricular outflow tract, ascending aorta diameter, left atrial diameter, septal thickness, left ventricular posterior wall thickness, left ventricular end-systolic diameter, left ventricular end-diastolic diameter, pulmonary artery diameter, right ventricular diameter, right atrial diameter, left ventricular ejection fraction and others.

### Diagnostic criteria for diastolic dysfunction

According to the detection methods recommended by the American Echocardiography Association and the European Association of Cardiovascular Imaging in 2016, the following 4 indicators were used to measure the disease:1. Lateral septal e velocity < 7 cm/s, or lateral wall e velocity < 10 cm/s; 2. Average E/e > 14; 3. Left atrial volume index > 34ml/m^2^; 4. Peak velocity of tricuspid regurgitation > 2.8 m/s [5]. If two or more indicators did not exceed the critical value, it indicated normal diastolic function; if two or more indicators exceed the critical value, it indicated abnormal diastolic function. If there were exactly two indicators reaching the critical value, it should be judged according to the clinical manifestations.

In the meantime, we conducted a questionnaire survey on these patients, it consists of three questions:the symptoms of chest tightness, chest pain, shortness of breath after activity recorded in the past 3 months. The questionnaire was filled out at the same time when the patient underwent the echocardiography. Two hemodialysis specialist nurses conducted the questionnaire beside the patients while the echocardiography was completed. Even if it is missed sometimes, it will be filled in 1–2 days later.All patients signed an informed consent form before the echocardiography examination.

### Statistical analysis

SPSS statistical software was used for analysis in the study(version 20.0;IBM Corp.Armonk,NY,USA). The measured data conforming to normal distribution were expressed as mean ± standard deviation, and comparison between groups was performed by t-test. The measured data of non-normal distribution were represented as M (Q1,Q3), and comparison between two groups was performed by rank sum test. The adoption rate or percentage of enumeration data was expressed, and comparison between groups was performed by chi-square test. Binary Logsitic regression analysis was used to screen the risk factors for LVDD. The results were expressed as odds ratio (OR), and *p* < 0.05 was considered statistically significant.

## **Results**

**Fig. 1:**
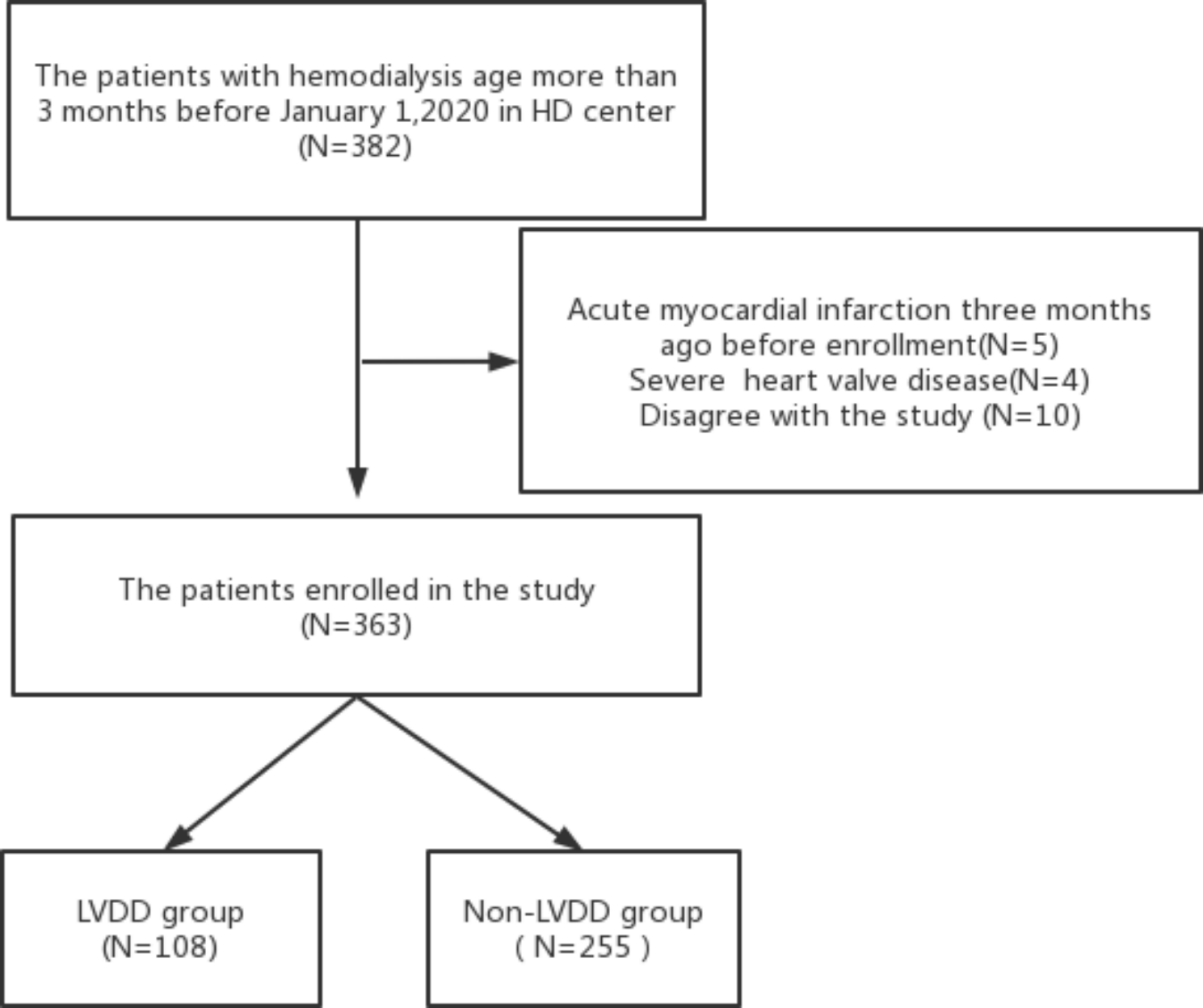
The flow chart for enrollment process of MHD patients in the study.MHD,maintenance hemodialysis.LVDD,left ventricular diastolic dysfunction

1. As shown in Fig. 1, a total of 363 MHD patients were included, including 108 (29.8%) LVDD patients and 137 were male. Patients in the LVDD group were older than those in the non-LVDD group (*p<*0.05). The serum BNP and cTnI levels were higher (*p<*0.05), and the triglyceride levels were lower (*p* = 0.043) in patients of LVDD group. Furthermore, the proportion of LVDD group combined with coronary heart disease increased, and the symptoms of chest tightness and shortness of breath more likely occurred in LVDD patients (all *p<*0.05)(Table 1).

2. Subgroup analysis of age. According to age, MHD patients were divided into young group (*<*40 years old), middle-aged group (40–60 years old) and elderly group (> 60 years old). As shown in Table 1, with the gradual increase of age, the proportion of patients in LVDD group increased gradually, which were 4.6%, 27.8% and 67.6%, respectively, and the difference was statistically significant (*p* = 0.002) (Table 1).

**Table 1:** Comparison of basic characteristics of MHD patients with and without diastolic dysfunction [x ± s, N (%)]

Variable	Non-LVDD group (N = 255)	LVDD group (N = 108)	p-value
Male	137(67.8%)	65(60.2%)	0.257
Age	58.13 ± 14.46	64.26 ± 12.35	< 0.001
**Age group**			0.002
Young group	33 (12.9%)	5 (4.6%)	
Middle-aged group	98 (38.4%)	30 (27.8%)	
Elderly group	124 (48.6%)	73 (67.6%)	
Dialysis age(month)	53.12 ± 37.84	49.23 ± 38.80	0.373
Average ultrafiltration capacity(ml)	2145 ± 801.9	2095 ± 784.43	0.584
Hypertension	194 (76.1%)	88 (81.5%)	0.258
Heart failure	126 (49.4%)	65 (60.2%)	0.06
Coronary heart disease	49 (19.2%)	35 (32.4%)	0.006
Chest distress	85 (33.3%)	52 (48.1%)	0.008
Anhelation	75 (29.4%)	48 (44.4%)	0.006
Chest pain	24 (9.4%)	16 (14.8%)	0.133
Hemoglobin(g/L)	108.62 ± 13.72	110.6 ± 13.33	0.206
Serum creatinine(umol/L)	1019.22 ± 251.63	983.69 ± 289.97	0.241
Uric acid(umol/L)	461.42 ± 105.94	448.62 ± 88.45	0.273
Albumin(g/L)	40.35 ± 3.50	39.81 ± 3.04	0.168
Blood sugar(mmol/L)	10.34 ± 5.84	10.49 ± 5.75	0.818
cTnI(ug/L)	0.00 ± 0.06	0.11 ± 0.74	0.022
Parathormone(pmol/L)	56.23 ± 47.07	61.89 ± 55.11	0.321
Phosphorus(mmol/L)	2.19 ± 0.64	2.19 ± 0.629	0.966
Calcium(mmol/L)	2.09 ± 0.28	2.07 ± 0.33	0.719
BNP(pg/ml)	480.89 ± 850.81	1041.06 ± 1470.80	<0.001
Triglyceride(mmol/L)	2.1 ± 1.64	1.75 ± 1.05	0.043
High-density lipoprotein(mmol/L)	1.06 ± 0.26	1.09 ± 0.29	0.334
Low density lipoprotein(mmol/L)	2.08 ± 0.77	2.32 ± 2.17	0.119
Total cholesterol(mmol/L)	3.96 ± 1.30	4.01 ± 0.97	0.707

Abbreviations: *cTnI* cardiac troponin I, *BNP* brain natriuretic peptide

3. Comparison of echocardiographic parameters between the two groups. As shown in Table 2, LA, IVS, LVPW, LVDd, LVDs and PA in LVDD group were all higher than those of the non-LVDD group, while FS was lower (all *p* < 0.05). Meanwhile, the proportion of left ventricular hypertrophy, left heart enlargement, systolic dysfunction, valve regurgitation, valve stenosis, pulmonary hypertension and pericardial effusion increased significantly (*p* < 0.05), and the ejection fraction of LVDD group was lower than that of non-LVDD group (*p* = 0.001).

**Table 2:** Comparison of echocardiographic parameters in MHD patients with and without diastolic dysfunction

Variable	Non-LVDD group (N = 255)	LVDD group (N = 108)	P-value
RVOT (mm)	22.78 ± 13.05	22.79 ± 3.69	0.993
AO (mm)	26.54 ± 19.33	26.59 ± 3.21	0.978
LA (mm)	28.96 ± 5.39	32.24 ± 6.35	<0.001
IVS (mm)	11.25 ± 1.76	12.26 ± 1.76	<0.001
LVPW (mm)	10.51 ± 1.48	11.26 ± 1.97	<0.001
LVDd (mm)	42.02 ± 6.747	46.24 ± 8.38	<0.001
LVDs (mm)	26.80 ± 5.89	34.27 ± 27.9	<0.001
PA (mm)	20.78 ± 2.25	21.62 ± 2.38	0.002
RV (mm)	26.4 ± 14.02	26.06 ± 5.19	0.807
RA (mm)	28.01 ± 4.08	28.10 ± 5.60	0.858
FS (%)	36.42 ± 6.16	34.48 ± 8.01	0.013
LVH	118(46.3%)	70(64.8%)	0.001
Left ventricular enlargement	11(4.3%)	15(13.9%)	0.001
Systolic dysfunction	14(5.5%)	23(21.3%)	0.000
Valvular regurgitation	158(62%)	95(88%)	0.000
Valvular stenosis	2(0.8%)	5(4.6%)	0.015
Ejection fraction (%)	65.42 ± 9.39	61.23 ± 12.63	0.001

Abbreviations: *RVOT* right ventricle outflow tract,*AO* aorta,*LA* left atrial,*RA* right atrial,*IVS* interventricular septal thickness,*LVPW* left ventricular posterior wall,*LVDd* left ventricular end diastolic dimension,*LVDs* left ventricular end-systolic dimension,*PA* pulmonary artery,*RV* right ventricular diastolic diameter,*FS* fractional shortening,*LVH* left ventricular hypertrophy,Systolic dysfunction defined as ejection fraction less than 40%

4. Risk factors analysis for LVDD in MHD patients. Logistic regression analysis showed that age, coronary heart disease, left ventricular hypertrophy, recent chest tightness and shortness of breath were significantly associated with diastolic dysfunction in MHD patients, as shown in Table 3. Multivariate logistic regression analysis showed that age and left ventricular hypertrophy were independent risk factors for LVDD. Compared with young MHD patients aged less than 40 years, elderly MHD patients aged more than 60 years old had a significantly higher risk of LVDD (OR = 3.86, 95% CI 1.429–10.429). Meanwhile, left ventricular hypertrophy was also significantly associated with LVDD (OR = 2.227, 95%CI 1.383–3.586) (Table 4).

**Table 3:** Univariate logistic regression analysis of MHD patients with diastolic dysfunction

Risk factors	P-value	OR	95%CI
**Age group**			
Young group		1	
Middle-aged group	0.179	2.02	0.724 ~ 5.635
Elderly group	0.007	3.885	1.452 ~ 10.394
Coronary heart disease	0.007	2.016	1.211 ~ 3.354
Left ventricular hypertrophy	0.001	2.139	1.343 ~ 3.406
Chest distress	0.008	1.857	1.174 ~ 2.938
Anhelation	0.006	1.92	1.206 ~ 3.058

**Table 4:** Multivariate logistic regression analysis of MHD patients with diastolic dysfunction

Risk factors	P-value	OR	95%CI
**Age group**			
Young group		1	
Middle-aged group	0.235	1.873	0.665 ~ 5.28
Elderly group	0.008	3.86	1.429 ~ 10.429
Left ventricular hypertrophy	0.001	2.227	1.383 ~ 3.586

**Abbreviations**:*OR* odds ratio

## Discussion

Cardiovascular disease is the most common cause of death in MHD patients [2]. LVDD is an early structural and functional change in the heart, mainly manifested as impaired ventricular filling, abnormal left ventricular relaxation in early diastole and reduced myocardial compliance [6]. When these changes are gradually aggravated, resulting in irreversible abnormalities of myocardial structure, heart failure will develop, which can lead to poor prognosis [7]. LVDD will significantly increase the risk of death in MHD patients [3].Meanwhlie, LVDD is more closely related to physical function and body composition than left ventricular systolic dysfunction in MHD patients [8].Due to the lack of obvious symptoms and timely diagnosis, it is very important to identify the risk factors of LVDD and carry out early intervention.

Previous studies have shown that increasing age is significantly associated with the development of diastolic dysfunction [9]. The prevalence of LVDD was 36% (15.8-52.8%) in the elderly over 60 years old and 51.3% in the elderly over 80 years old [10]. In this study, patients were divided into three groups: young, middle-aged and elderly. The proportion of middle-aged and elderly patients (aged over 60 years) in LVDD group was 67.6%, which was significantly higher than that in the young group (4.6%) (*p*<0.05). Multivariate logistic regression also showed that age was an independent risk factor for LVDD, and the risk of LVDD in the elderly group was 3.86 times higher than that of the young group (95%CI 1.429–10.429). The growth of the age is also an important risk factor for cardiovascular disease. With the increase of age, the formation of reactive oxygen species increases, and mitochondria fusion/fission imbalances, such as continuous myocardial cell stress, causes irreversible damage to the myocardial cells. Meanwhile, vascular wall stiffness, left ventricular wall thickness and fibrosis increase with age, which are the pathophysiological basis for cardiac structural changes and electrophysiological dysfunction [11]. In this study, LVH was also an independent risk factor for LVDD in MHD patients (OR = 2.227, 95%CI 1.383–3.586). LVH is one of the most common myocardial changes in patients with end-stage renal disease, and is often associated with myocardial fibrosis and diastolic dysfunction [12]. LVH is an independent risk factor for cardiovascular events in MHD patients, and dialysis patients with LVH are prone to myocardial ischemia, heart failure and sudden cardiac death [13]. CKD-related risk factors can easily lead to cardiac fibrosis in uremic patients, structural abnormalities such as cardiac hypertrophy, myocardial fibrosis, and thickening of intramural arteries and arterioles [15]. In addition, the adaptive response to pressure and volume overload lead to cardiac hypertrophy and vascular remodeling [14]. These changes make the heart of patients with end-stage renal disease prone to diastolic dysfunction. In addition, the development of MHD will result in hemodynamic changes caused by transient myocardial ischemia and low blood pressure, and myocardial suppression [15]. LVDD is present in patients who have ventricular filling obstacle. During dialysis ultrafiltration, the reduction of blood volume leads to the reduction of cardiac output, and the change of blood pressure causes insufficient myocardial perfusion, leading to myocardial damage, fibrosis, myocardial hypertrophy and diastolic dysfunction [16]. In this study, we also proved that compared with the non-LVDD group, the LVDD group had a significantly higher proportion of left heart enlargement, systolic dysfunction, valve regurgitation, valve stenosis, pulmonary hypertension and pericardial effusion (*p* < 0.05), while the LVDD group had a lower ejection fraction (*p* = 0.001).

Since the diastolic dysfunction may last for years before any symptoms appear and it may represent the first stage of diastolic heart failure, it is important to find diastolic dysfunction early and start treatment before irreversible structural changes and systolic dysfunction occurring [4]. Previous studies have shown that anemia, hypoalbuminemia, calcium and phosphorus metabolism disorders and other pathological conditions are important influencing factors of LVDD, and these conditions have a high incidence in CKD patients [16, 17]. Therefore, for MHD patients, correcting anemia, improving nutritional status and calcium and phosphorus metabolism disorders will help to improve the prognosis of patients.

### Limits

This study was limited by its cross-sectional design, which did not allow causal conclusions to be drawn. And this study did not include long-term follow-up of LVDD patients to further examine the risk of cardiovascular events and all-cause death in MDH patients. In addition, LVDD was not graded in the analysis of diastolic dysfunction. In future studies, prospective stratified studies of LVDD patients are needed to explore treatment methods to improve diastolic dysfunction.

## Conclusion

LVDD is a common complication in MHD patients, but the increased risk of death is easily ignored. Age and left ventricular hypertrophy are independent risk factors for LVDD in MHD patients. Early detection and intervention of LVDD may be beneficial to reduce the incidence of cardiovascular events.

## Data Availability

The datasets used in this article and/or analysed during the current study are available from the corresponding author or the first author on reasonable request.

## Abbreviations

MHD: Maintenance hemodialysis
LVDD: Left ventricular diastolic dysfunction
CVD: Cardiovascular disease
CKD: Chronic kidney disease
cTnI: Cardiac troponin I
BNP: Brain natriuretic peptide
RVOT: Right ventricle outflow tract
AO: Aorta
LA: Left atrial
RA: Right atrial
IVS: Interventricular septal thickness
LVPW: Left ventricular posterior wall
LVDd: Left ventricular end diastolic dimension
LVDs: Left ventricular end-systolic dimension
PA: Pulmonary artery
RV: Right ventricular diastolic diameter
FS: Fractional shortening
LVH: Left ventricular hypertrophy

## References

[1] GBD Chronic Kidney Disease Collaboration. Global,regional, and national burden of chronic kidney disease,1990–2017: a systematic analysis for the global burden of Disease Study 2017 [J].Lancet, 2020, 395(10225):709–33. DOI:10.1016/S0140-6736(20)30045-3. 10.1016/S0140-6736(20)30045-3 PMC7049905 32061315

[2] House AA, Wanner C, Sarnak MJ (2019). Heart failure in chronic kidney disease: conclusions from a kidney disease: improving global outcomes (KDIGO) Controversies Conference. Kidney Int.

[3] De Lima JJG, Macedo TA, Gowdak LHW et al. Diastolic and systolic left ventricular dysfunction and mortality in chronic kidney disease patients on haemodialysis. Nephrology (Carlton). 2022 Jan;27(1):66–73. doi: 10.1111/nep.13960. Epub 2021 Aug 19. PMID: 34378284. 10.1111/nep.13960 34378284

[4] Ogawa T, Koeda M, Nitta K. Left ventricular diastolic dysfunction in end-stage kidney disease: pathogenesis, diagnosis, and treatment. Ther Apher Dial. 2015 Oct;19(5):427–35. doi: 10.1111/1744-9987.12301. Epub 2015 Apr 27. PMID: 25916171. 10.1111/1744-9987.12301 25916171

[5] Nagueh SF, Smiseth OA, Appleton CP, et al. Recommendations for the evaluation of left ventricular diastolic function by Echocardiography: an update from the American Society of Echocardiography and the European Association of Cardiovascular Imaging. Eur Heart J Cardiovasc Imaging. 2016 Dec;17(12):1321–60. 10.1093/ehjci/jew082. Epub 2016 Jul 15. PMID: 27422899. 10.1093/ehjci/jew082 27422899

[6] Deswal A. Diastolic dysfunction and diastolic heart failure: mechanisms and epidemiology. Curr Cardiol Rep. 2005 May;7(3):178 – 83. doi: 10.1007/s11886-005-0074-7. PMID: 15865857. 10.1007/s11886-005-0074-7 15865857

[7] HILL J A,OLSON E N.Cardiac plasticity[J]. N Engl J Med 2008 Mar 27; 358 (13):1370–80.DOI: 10.1056/NEJMra072139. 10.1056/NEJMra072139 18367740

[8] Jeong JH, Wu PT, Kistler BM, et al. The presence and impact of diastolic dysfunction on physical function and body composition in hemodialysis patients. J Nephrol. 2015 Dec;28(6):739–47. 10.1007/s40620-015-0188-y. Epub 2015 Mar 10. PMID: 25753450. 10.1007/s40620-015-0188-y PMC10413439 25753450

[9] Bello H, Norton GR, Peterson VR, et al. Hemodynamic determinants of Age Versus Left ventricular diastolic function relations across the full adult age range. Hypertension. 2020 Jun;75(6):1574–83. 10.1161/. HYPERTENSIONAHA. 119.14622. Epub 2020 Apr 6. PMID: 32248702. 10.1161/HYPERTENSIONAHA.119.14622 32248702

[10] van Riet EE, Hoes AW, Wagenaar KP et al. Epidemiology of heart failure: the prevalence of heart failure and ventricular dysfunction in older adults over time. A systematic review. Eur J Heart Fail. 2016 Mar;18(3):242 – 52. doi: 10.1002/ejhf.483. Epub 2016 Jan 4. PMID: 26727047. 10.1002/ejhf.483 26727047

[11] Henning RH, Brundel BJJM. Proteostasis in cardiac health and disease. Nat Rev Cardiol. 2017 Nov;14(11):637–653. doi: 10.1038/nrcardio.2017.89. Epub 2017 Jun 29. PMID: 28660894. 10.1038/nrcardio.2017.89 28660894

[12] Hsu CW, WengCH,Lee CC et al. Association of low serum aluminum level with mortality in hemodialysis patients [J].Ther Clin ï¼²isk Manag, 2016, 12(6) : 1417-1424.DOI: 10.2147/TCRM.S113829. eCollection 2016. 10.2147/TCRM.S113829 PMC5028174 27695338

[13] Tyralla K. Amann K.Morphology of the heart and arteries in renal failure.Kidney Int 2003;(Suppl 84):S80-3 DOI: 10.1046/j.1523-1755.63.s84.1.x. PMID: 12694316. 10.1046/j.1523-1755.63.s84.1.x 12694316

[14] Unger ED, Dubin RF, Deo R, et al. Association of chronic kidney disease with abnormal cardiac mechanics and adverse outcomes in patients with heart failure and preserved ejection fraction. Eur J Heart Fail. 2016 Jan;18(1):103–12. 10.1002/ejhf.445. Epub 2015 Dec 3. PMID: 26635076; PMCID: PMC4713321. 10.1002/ejhf.445 PMC4713321 26635076

[15] Daugirdas JT. Pathophysiology of dialysis hypotension: an update. Am J Kidney Dis. 2001 Oct;38(4 Suppl 4):S11-7. DOI: 10.1053/ajkd.2001.28090. PMID: 11602456. 10.1053/ajkd.2001.28090 11602456

[16] Malik J, Kudlicka J, Valerianova A et al. Diastolic dysfunction in asymptomatic hemodialysis patients in the light of the current echocardiographic guidelines. Int J Cardiovasc Imaging. 2019 Feb;35(2):313–317. doi: 10.1007 /s10554-019-01564-2. Epub 2019 Feb 27. PMID: 30815807. 10.1007/s10554-019-01564-2 30815807

[17] García-Bello JA, Ortiz-Flores J, de la Torres FE et al. Anemia and hypoalbuminemia as risk factors for left ventricular diastolic dysfunction in children with chronic kidney disease on peritoneal dialysis. Nefrologia (Engl Ed). 2018 Jul-Aug;38(4):414–419. English, Spanish. doi: 10.1016/j.nefro.2017.11.024. PMID: 30032857. 10.1016/j.nefro.2017.11.024 30032857

